# Development and validation of a scatter-corrected CBCT image-guided method for cervical cancer brachytherapy

**DOI:** 10.3389/fonc.2022.942016

**Published:** 2022-10-27

**Authors:** Ailin Wu, Hehe Cui, Xiao Jiang, Bing Yan, Aidong Wu, Yunqin Liu, Lei Zhu

**Affiliations:** ^1^ Department of Radiation Oncology, The First Affiliated Hospital of University of Science and Technology of China (USTC), Division of Life Sciences and Medicine, University of Science and Technology of China, Hefei, China; ^2^ Department of Engineering and Applied Physics, University of Science and Technology of China, Hefei, China

**Keywords:** cervical cancer, CBCT, scatter correction, model-based dose calculation algorithm, brachytherapy

## Abstract

**Background and purpose:**

Multiple patient transfers have a nonnegligible impact on the accuracy of dose delivery for cervical cancer brachytherapy. We consider using on-site cone-beam CT (CBCT) to resolve this problem. However, CBCT clinical applications are limited due to inadequate image quality. This paper implements a scatter correction method using planning CT (pCT) prior to obtaining high-quality CBCT images and evaluates the dose calculation accuracy of CBCT-guided brachytherapy for cervical cancer.

**Materials and methods:**

The CBCT of a self-developed female pelvis phantom and five patients was first corrected using empirical uniform scatter correction in the projection domain and further corrected in the image domain. In both phantom and patient studies, the CBCT image quality before and after scatter correction was evaluated with registered pCT (rCT). Model-based dose calculation was performed using the commercial package Acuros^®^BV. The dose distributions of rCT-based plans and corrected CBCT-based plans in the phantom and patients were compared using 3D local gamma analysis. A statistical analysis of the differences in dosimetric parameters of five patients was also performed.

**Results:**

In both phantom and patient studies, the HU error of selected ROIs was reduced to less than 15 HU. Using the dose distribution of the rCT-based plan as the baseline, the γ pass rate (2%, 2 mm) of the corrected CBCT-based plan in phantom and patients all exceeded 98% and 93%, respectively, with the threshold dose set to 3, 6, 9, and 12 Gy. The average percentage deviation (APD) of D_90_ of HRCTV and D_2cc_ of OARs was less than 1% between rCT-based and corrected CBCT-based plans.

**Conclusion:**

Scatter correction using a pCT prior can effectively improve the CBCT image quality and CBCT-based cervical brachytherapy dose calculation accuracy, indicating promising prospects in both simplified brachytherapy processes and accurate brachytherapy dose delivery.

## 1 Introduction

Cervical cancer, the fourth most common cancer among women, is a worldwide disease with high incidence and mortality rates, especially in developing countries (1, 2). Benefiting from the steep dose curves and the short source-to-tumor distance, brachytherapy (BT) can deliver an ultrahigh dose to the target volume with maximal preservation of the organs at risk (OARs). Therefore, BT is considered essential to conventional radiotherapy for cervical cancer, and previous research reported that BT can significantly improve the local control rate of the tumor and the 5-year survival rate of patients in combination with external beam radiotherapy (EBRT) (3).

Despite the tremendous success, the inadequate rates of local control and recurrence still hamper the effectiveness of the treatment for advanced cervical cancer (4, 5). Further improvement in the patient survival rate requires more accurate dose delivery. Current BT dose delivery is usually compromised by two key factors. One is the error of the commonly used water-based dose calculation recommended by AAPM TG-43U1 (6). This dose estimation strategy omits the human tissue heterogeneity and the effect of the applicator with high-Z materials, and the difference in back scatter between the human body and water is not considered. As revealed in AAPM TG-186 (7), differences between water-based and media-based dose calculations may exceed a factor of 10 in specific situations. Therefore, the use of model-based dose calculation (MBDC) is advocated for clinical practice to promote dose calculation accuracy (8). Since MBDC makes use of tissue composition and mass density as estimated from CT images of BT patients, high-quality images are considered the essential assurance for accurate dose calculation.

The second adverse factor is patient organ variations and applicator displacement due to multiple transfers and long waits (9, 10). Since cumbersome x-ray/CT/MR machines have not been widely installed in the BT treatment room, patient transfers between the imaging room and the treatment room are inevitable, which usually not only increases patient suffering but also causes organ variations and applicator displacement. During scanning, planning, and treatment, this inconsistency in the patients’ anatomy induces a large dose delivery error since the dose is sharply decreased (11). Due to its advantages, including low cost, volume imaging, and simple integration, cone-beam CT (CBCT) is promising for resolving all the above adverse factors. Provided a CBCT system is installed in the treatment room, the CBCT images obtained before treatment can provide consistent patient anatomy and applicator positions as those during BT dose delivery. More importantly, the calibrated physical quantities from CT numbers, such as electron density or Z-number (12, 13), can be used for MBDC. In this case, the applicator placement/adjustment and MBDC and BT dose delivery can be completed all in one room, indicating a much simplified treatment process, shortened treatment time, and improved patient comfort.

However, the severe photon scatter, a general CBCT issue (14, 15), unavoidably degrades the soft-tissue contrast and introduces large CT number bias, which consequently results in inaccurate organ delineation and dose calculation (16, 17). It is thus seen that effective and efficient scatter correction is critical for CBCT-based radiotherapy techniques. Various CBCT scatter corrections have been developed during the last two decades (18–22), which enhances its utility in dose calculation for adaptive EBRT planning (23). Recently, on-site CBCT has attracted increasing attention in adaptive BT (24). However, existing study results show that the current CBCT image quality is not adequate to meet the clinical requirements (24–26), and performance improvements of CBCT images are needed. Recall that cervical BT is always coupled with EBRT (3), and the prior information-based methods (27, 28) are especially suitable in BT since high-quality EBRT-CT routinely obtained for treatment planning can be used as a prior. However, the existing prior information-based methods are only carried out in the projection domain or in the image domain. The performance of those methods may be degraded when the CBCT is obtained under poor conditions.

In this work, we propose hybrid-domain CBCT scatter correction using EBRT-CT as a prior. A self-developed BT phantom is used in the first study, and the quantitative image analysis and accuracy evaluation of the dose calculation are carried out. The patient study presents the comparison of image quality and clinical dose assessment based on the rCT and scatter-corrected CBCT images, which validates the clinical feasibility of scatter-corrected CBCT image-guided brachytherapy (IGBT).

## 2 Materials and methods

### 2.1 Hybrid-domain scatter correction

**Figure 1:**
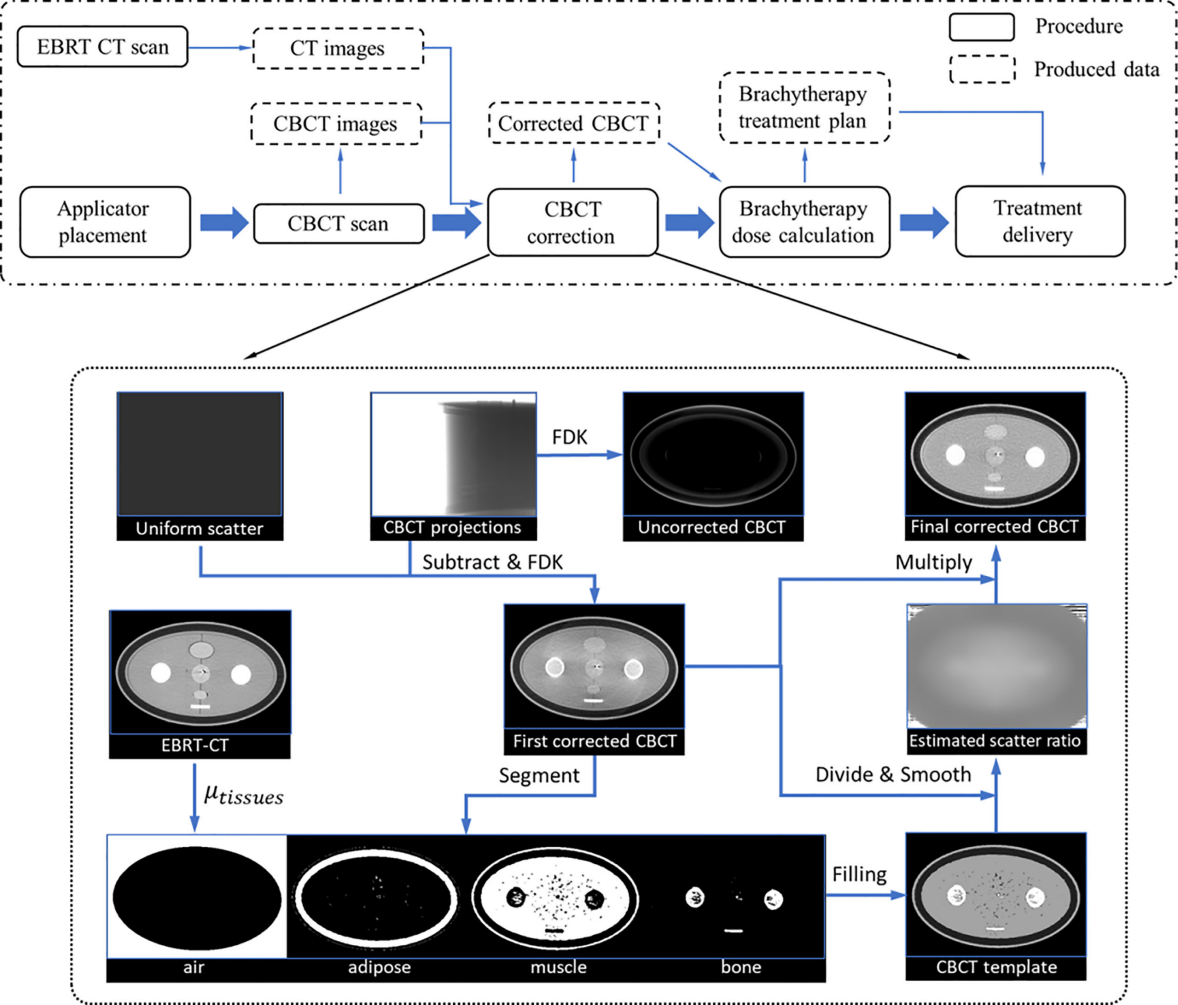
Dot-dash boxes: the flowchart of CBCT-based cervical brachytherapy. Dotted box: detailed workflow of the scatter correction.

In the conventional IGBT process, the patient was first implanted with applicators in the gynecological room, then transferred to the x-ray/CT/MRI room to acquire images for treatment planning and dose calculation, and finally, the patient needs to be transferred to the BT room for treatment. However, this complex treatment process, coupled with the long wait times, inevitably caused applicator displacement and tissue variation. Because of a large dose gradient around the radiation source, even a small deviation can result in an unacceptable dose change to the tumor and OARs. As an ideal solution, CBCT IGBT was proposed, which realized applicator insertion, imaging, and treatment delivery in the same room. To ensure that CBCT images can not only meet the requirements of target delineation but also achieve accurate dose calculation, we put forward hybrid-domain CBCT scatter correction for BT. The workflow of this method is depicted in **Figure 1**. For simplicity, CT images acquired during the EBRT/BT are referred to as EBRT/BT-CT. To improve the HU accuracy of CBCT, we implemented a hybrid-domain scatter correction using EBRT-CT as a prior, which is illustrated in the dotted box of **Figure 1**, with each step summarized as follows:

**Step 1**: Obtain the linear attenuation coefficients (LAC) of four tissues (air, fat, muscle, and bone) from EBRT-CT, and their values are abbreviated as ${\bar{\mu }}_{air},{\bar{\mu }}_{fat},{\bar{\mu }}_{muscle}\;,\text{and }{\bar{\mu }}_{bone}$ . Because of the high resolution and uniformity of EBRT-CT, the HU number of different tissues (air, adipose, muscle, and bone) can be easily distinguished in the CT histogram distribution. The mode of each tissue is obtained according to the histogram and used as the mean value of each tissue. Note that the directly obtained value is the CT number value with HU units and the LAC is then calculated based on Equation (1).


(1) $$\mu_{m}=\frac{CT\;number}{1000}\times \mu_{w}+\mu_{w}$$

*μ_m_* and *μ_w_* represent the LAC of the material and water, respectively.

**Step 2**: Generate the first-pass scatter-corrected projections (*p_c_*) by subtracting an empirical uniform scatter $\left(\bar{s}\right)$ from the raw CBCT projections (*p_raw_*). Then, the CBCT image (*CBCT_c_*) is reconstructed using *p_c_ via* the Feldkamp–Davis–Kress (FDK) algorithm.

**Step 3**: The *CBCT_c_* is segmented into four parts: air, fat, muscle, and bone, and the template image (*CBCT_t_* ) is generated by filling each part with the referenced LAC. Then, *CBCT_t_* is created using the LAC from EBRT-CT.

**Step 4**: The scatter ratio (*r*) is first calculated as *r = CBCT_t_*/*CBCT_c_* , and then a binary mask (*f*) is generated to sample the low-frequency signals on *r*:


(2) $$f=\left\{ \begin{array}{l} 0,\;|r|>r_{\max}or|\nabla r|\;>\;G_{\max}\\ 1,\;otherwise \end{array}\right.$$

*r_max_* and *G_max_* are the maximum intensity and the gradient of the scatter ratio, respectively. Their value is chosen such that 80% of all pixels is smaller than the chosen value.

**Step 5**: Sparse-sampled scatter ratio *r* is further smoothed and extended to the whole images *via* a local filtration technique (29):


(3) $$r_{f}=\frac{(r\cdot f)**w}{f**w}$$

where ** represents the 2D convolution operation and *w* and *r_f_* denote the Gaussian filter and the smoothed scatter ratio, respectively.

**Step 6**: Obtain the final corrected CBCT (*CBCT_fc_*) by scaling the *CBCT_c_* by the smoothed ratio (*r_f_*).

The CBCT images reconstructed from raw projections suffer severe nonuniformity, which hampers accurate tissue differentiation. To resolve this, an empirical uniform scatter correction is firstly performed in the projection domain to roughly alleviate the nonuniformity and facilitate segmentation. The uniform scatter correction method is inspired by the low-frequency feature of the scatter signal. In this method, a constant value is considered the scatter signal, and the corrected projection is generated by subtracting the value from the raw projection. In this work, the constant value is set such that 90% of the object projection signals are larger, and a soft-cut algorithm currently used in previous studies is adopted to ensure the corrected projection signal positivity (22). *CBCT_c_* is reconstructed using the corrected projections *via* FDK.

The CBCT segmentations are generated by transferring the EBRT-CT segmentation *via* the commercial software MIM (software version 7.1.2; MIM Software Inc., Cleveland, OH, USA). The segmentation of EBRT-CT is performed based on the threshold. The CT number of EBRT-CT in the range [−1,024 −500], [−125 −60], [15 85], and >190 HU is thought to be air, adipose, muscle, and bone tissues, respectively. Although the EBRT-CT is of high quality, the boundary between the two tissues is not easily distinguished automatically; the tissue whose CT number is not within the above interval was considered to be air. Then, the value of those values in the binary mask *f* is equal to 0, which means that the weight of those values in calculating the smoothed ratio *r_f_* is 0.

Since the applicator used in this study is made of high-Z titanium, its HU value is much larger than that of human tissues. Considering that the applicator is not deformed during treatment, we performed a separate CT scan for the applicator and segmented it as the applicator ground truth. Then, the applicator in *CBCT_fc_* is segmented separately, and the applicator in the CT images is rigidly registered and transferred to the final corrected CBCT.

### 2.2 Data acquisition and processing

**Figure 2:**
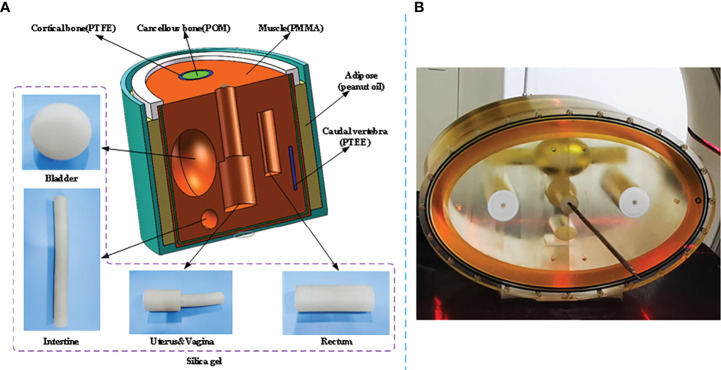
Illustration of the self-developed female pelvis BT phantom. **(A)** Structure diagram and materials of the phantom. **(B)** Pelvis phantom during a real CT scan.

A self-developed female pelvis BT phantom is shown in **Figure 2**. The phantom dimension and organ position were determined based on Asian female patients with an elliptical cross-sectional area of 340 mm * 200 mm. The materials with similar CT numbers to the corresponding organs or tissues were selected to mimic the real female pelvis. As shown in **Figure 2A**, the molds of OARs, i.e., the bladder, intestine, and rectum, were made up of silica gel, peanut oil, polymethyl methacrylate (PMMA), polyoxymethylene (POM), and polytetrafluoroethylene (PTFE) were used to simulate adipose, muscle, cortical bone, and cancellous bone respectively. The uterus and vagina were connected with a 2-mm-diameter elastic channel in it to enable applicator movement. In the phantom study, a tandem applicator (AL07522000; Varian Medical Systems, Palo Alto, CA) was inserted. **Figure 2B** displays the real pelvis phantom with the inserted applicator. A phantom study was used to demonstrate the feasibility of the proposed method.

To further evaluate the proposed method in clinical applications, five patients with stage IIB–IIIB cervical cancer were selected for retrospective analysis. These patients received EBRT and image-guided radiotherapy (IGRT) boost in combination with 3D high-dose-rate (HDR) BT (30) in Anhui Provincial Cancer Hospital. Before each BT patient received EBRT, a BT-CBCT scan was performed after the BT-CT scan to confirm the position of applicators. During this procedure, an effective external immobilization and a 3D transfer bed were used. The patient transfer was performed by multiple staff members in a coordinated effort to minimize the applicator displacement. A titanium Fletcher-Suit Delclos (FSD)-Style Applicator Set (AL13030001; Varian Medical Systems, Palo Alto, CA) was used for implantation, along with gauze packing.

In both phantom and patient studies, the CT images (including EBRT-CT and BT rCT) were acquired by a 16-slice CT machine (Discovery CT590 RT, GE). The CT machine operated in standard pelvis mode and reconstructed images with a size of 512 * 512 * 114 voxels, and the voxel size was 1.17 mm * 1.17 mm * 2.5 mm. The CBCT scan of the pelvis phantom was acquired in a tabletop CBCT system at the University of Science and Technology of China. The geometry of this system exactly matches that of a Varian On-Board Imager (OBI) CBCT system. To comprehensively evaluate the performance on scatter correction, the bowtie filter that can alleviate photon scatter was not installed in this system. The tube voltage, tube current, and pulse width were set to 125 kVp, 30 mA, and 10 ms, respectively. The patient CBCT scan was acquired on the commercial OBI system installed on Trilogy machine (Varian Medical Systems, Palo Alto, CA). The x-ray tube was operated at 125 kVp and 80 mA, with a bowtie filter mounted at the tube exit. Projection data were exported from the OBI CBCT system *via* the tabletop computer, and then the reconstructions were performed in MATLAB with and without scatter correction to obtain corrected CBCT and raw CBCT, respectively. The reconstructed volume had a size of 512 * 512 * 160 voxels, with a voxel size of 1 mm * 1 mm * 1 mm. Additionally, the rCT images and the corrected CBCT images were registered by using the MIM-Mastro DIR algorithm and the reg refine tool. For computation acceleration, FDK and the local filtration were implemented using CUDA C (NVIDIA, Santa Clara, CA).

### 2.3 Dose calculation

The rCT and *CBCT_fc_* of the phantom and patients were input into the commercial software BrachyVision (vision 15.5, Varian Medical Systems, Inc., Palo Alto, CA, USA). In the phantom dose calculation, the size of the dose-calculated volume was chosen as 386 * 245 * 200 voxels (1 mm * 1 mm * 1 mm for each voxel), which completely covered the entire area of the phantom. Ten dwell positions were manually defined, with a step size of 5 mm and a dwell time of 30 s at each position. The dose distributions (RD_CT) were obtained with MBDC *via* the Acuros^®^BV algorithm based on AAPM report TG-186. Since the phantom has no deformation, the rCT and *CBCT_fc_* were well matched. Using the same settings in the rCT dose calculation, the dose distribution RD_cor was obtained on *CBCT_fc_*.

In the patient dose calculation, experienced oncologists first delineated the target volume and OARs on both rCT and CBCT_fc_, then the physicist performed the applicator reconstruction on rCT images. To meet the dose recommendations by the gynecological (GYN) GEC-ESTRO working group (31, 32), the physical dose objectives of BT were 5.5 Gy/F for HR-CTV while the D_2cc_ of OARs was less than 5.3 Gy/F on the bladder and 4.15 Gy/F on the rectum, sigmoid, and intestine. Based on the rCT images, MBDC was completed by inverse planning *via* the Acuros BV optimization method and obtained the dose distribution RD_CT. Finally, the spatial coordinates of applicators, the dwell times, and the relative positions of radiation sources of the rCT plans were input into the CBCT_fc_ plans. The obtained dose distribution in heterogeneous media was referred to as RD_cor. The dose matrix was set the same for both RD_CT and RD_cor while the size of the dose-calculated volume was chosen as 2.5 mm * 2.5 mm * 1 mm for each voxel.

### 2.4 Evaluation

#### 2.4.1 CBCT image quality

Scatter always leads to severe cupping artifacts, indicating a much lower CT number, especially in the central area of CBCT images. The mean CT number value in selected ROIs is used to reflect the image accuracy on different tissues and the root-mean-square error (RMSE) is used to quantify the overall imaging accuracy, which is calculated as:


(4) $$RMSE=\sqrt{\frac{1}{n}\sum_{i=1}^{n}{\left(\bar{HU_{cbct}^{i}}-\bar{HU_{CT}^{i}}\right)}^{2}}$$

where $\bar{HU_{cbct}^{i}}$ and $\bar{HU_{CT}^{i}}$ are the mean CT number of the *i*th ROI in *CBCT_fc_* and rCT, respectively. In addition to CT number deviation, a low-frequency scatter signal also causes nonuniformity and contrast loss, which can be characterized by spatial nonuniformity (SNU) and the contrast-to-noise ratio (CNR):


(5) $$SNU={\bar{HU}}_{max}-{\bar{HU}}_{min}$$

(6) $$CNR=\frac{\left|\bar{HU_{r}}-\bar{HU_{b}}\right|}{\sigma_{r}}$$

where ${\bar{HU}}_{max}$ and ${\bar{HU}}_{\min}$ are the maximal and minimum mean CT number of the same tissue among the selected ROIs, respectively. $\bar{HU_{r}}$ and $\bar{HU_{b}}$ are both the mean CT number in the selected ROI and background, and *σ_r_* the standard deviation (STD) inside the ROI.

#### 2.4.2 Dose distribution

In the phantom study, rCT and CBCTfc were strictly matched and shared an identical plan. Thus, phantom results were used to quantitatively evaluate the dose calculation accuracy. The 3D local γ -index (33) was first calculated under three different distance and dose difference criteria (δr, δD), i.e., (1%, 1 mm), (2%, 1 mm), and (2%, 2 mm). The dose distribution on the rCT was used as a reference, and the dose threshold was set to 3 Gy, 6 Gy, 9 Gy, and 12 Gy.

In patient studies, rCT was matched with the *CBCT_fc_* images by MIM software. Therefore, 3D gamma analysis with different criteria and dose thresholds was also performed to evaluate the local dose difference of patients between the RD_CT and RD_cor. In addition, the parameters commonly used in the clinical dosimetric evaluation were statistically analyzed, including D_90_ (minimal dose delivered to 90%) of HR-CTV and the minimum dose of the 2 cm^3^ of the volume (D_2cc_) received by the bladder, rectum, sigmoid, and intestine.

## 3 Results

### 3.1 Phantom study

#### 3.1.1 CBCT image quality

**Figure 3:**
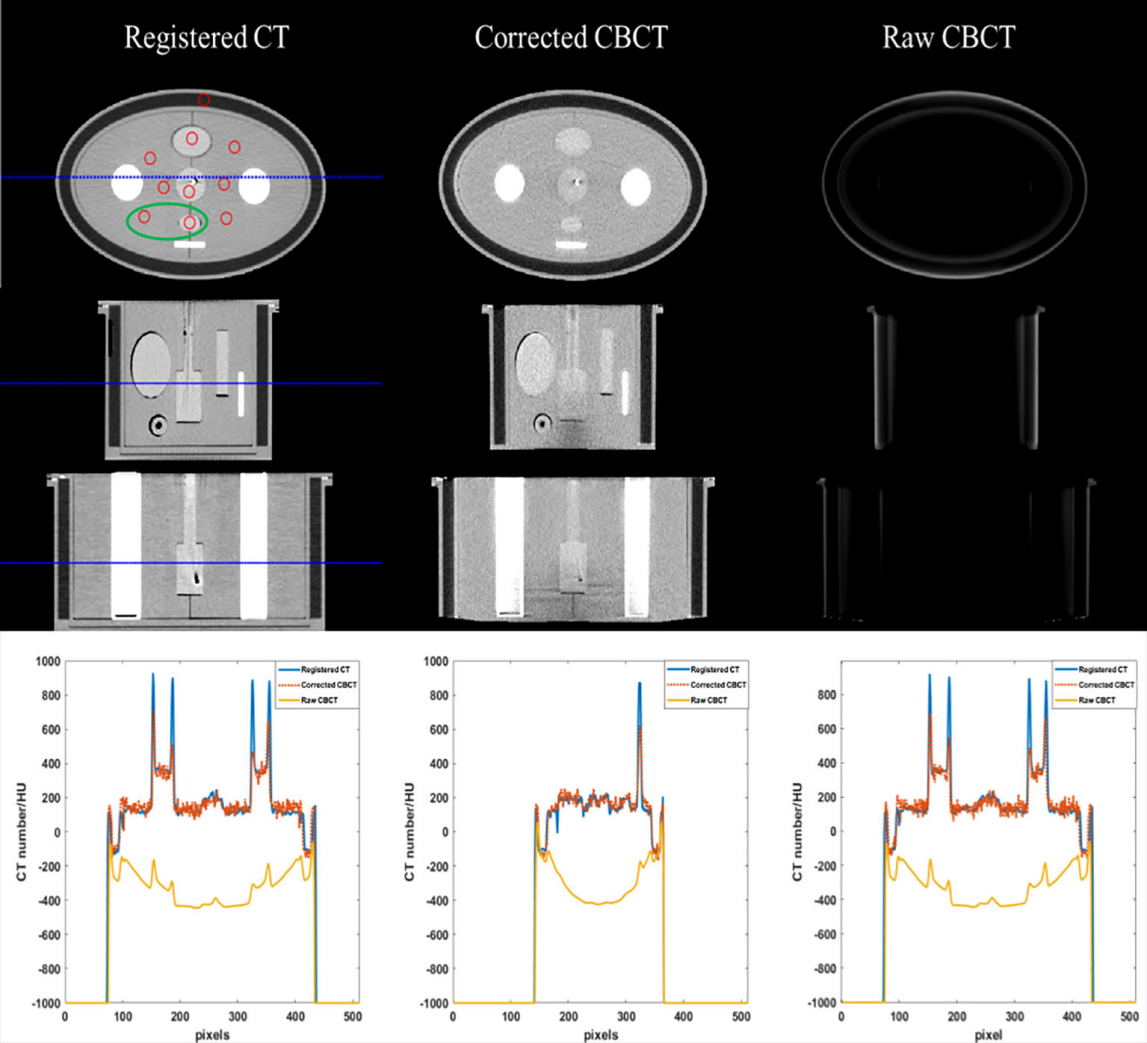
CT/CBCT images of the female pelvis phantom. The first three rows display the axial, sagittal, and coronal views from top to bottom; display window: [−200 300] HU. The mean CT number and SNU are calculated in the circle areas, and the CNR is calculated in the ellipse area. The 1D profiles indicated by the dotted lines are displayed in the last row.

**Figure 4:**
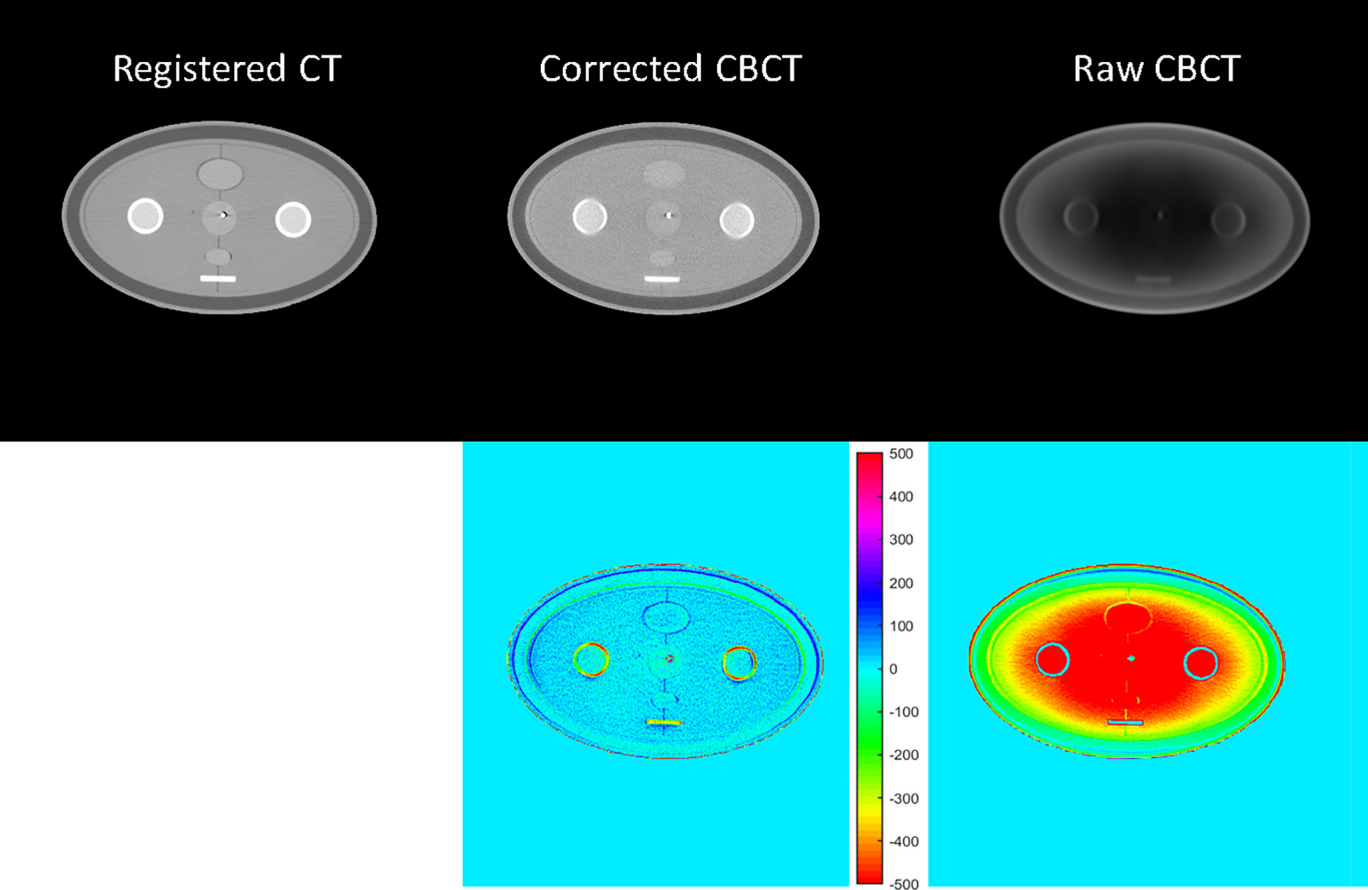
CT/CBCT images of the female pelvis phantom in the axial view and the CT number difference map of CBCT and CT; display window: [−500 500] HU.

Phantom images are displayed in **Figures 3** and **4**. Since a bowtie filter was not installed, raw CBCT images were severely contaminated by photon scatter such that no organ could be distinguished with the display window of [−200 300] HU, and the scatter-induced CT number error was spatially variant, as evidenced by the cupping 1D profiles in **Figure 3** and the pixel-level CT number difference of CBCT and CT in **Figure 4**. Although the raw CBCT seems better at the display window of [−500 500] HU in **Figure 4**, the image quality is not substantially improved. After the proposed correction, organs such as the uterus, intestine, rectum, and bladder were observed with the display window of [−200 300] HU, and the nearly coincident profiles indicated that the proposed method achieved accuracy comparable to that of rCT on the soft tissues.

The quantitative analysis shown in **Tables 1** and **2** further reveals the improvement in CT number accuracy. Excluding adipose tissue, each of the other nine ROIs suffered a CT number error of over 450 HU in the raw CBCT, which was reduced to less than 15 HU by the proposed correction. After scatter removal, the RMSE was reduced from 501 HU to less than 10 HU, the SNU was reduced to 16 HU from 107 HU and the CNR between the rectum and muscle was improved by a factor of 4.48. Although the improved CNR is still much lower than that of rCT, it is enough to differentiate the rectum from the background. Since the scatter removal amplifies the noise level (34), the standard deviation of CBCT corrected with the proposed method is larger than that in raw CBCT images.

**Table 1:** The CT number comparison of the 10 ROIs in **Figure 3** (unit: HU).

	Registered CT			Corrected CBCT				Raw CBCT			
		CT #	STD	Range	CT #	STD	Range	HU error	CT #	STD	Range	HU error
Muscle	134	9	(112, 156)	140	26	(76, 201)	6	−384	11	(−404, −363)	−518
	127	10	(98, 151)	126	25	(69, 214)	−1	−441	3	(−447, −436)	−569
	134	10	(109, 162)	142	26	(77, 228)	8	−353	13	(−377, −329)	−487
	125	10	(166, 211)	138	25	(141, 283)	13	−334	13	(−359, −309)	−459
	121	12	(160, 238)	129	27	(123, 253)	9	−435	2	(−438, −431)	−556
	131	9	(165, 231)	139	25	(126, 273)	9	−375	10	(−395, −353)	−505
Adipose	−112	10	(106, 151)	−108	40	(75, 192)	4	−128	41	(−178, 25)	−16
Bladder	191	10	(89, 148)	205	27	(63, 220)	14	−340	15	(−367, −307)	−532
Uterus	196	15	(112, 154)	181	24	(72, 216)	−15	−416	4	(−431, −407)	−612
Rectum	199	10	(−133, −49)	199	28	(−177, 86)	0	−380	11	(−403, −355)	−579

STD, standard deviation.

**Table 2:** RMSE, SNU, and CNR in the images of the female phantom.

	Registered CT	Corrected CBCT	Raw CBCT
RMSE (HU)	N/A	9	510
SNU (HU)	13	16	107
CNR	6.96	2.24	0.50

#### 3.1.2 Dose calculation precision

**Figure 5:**
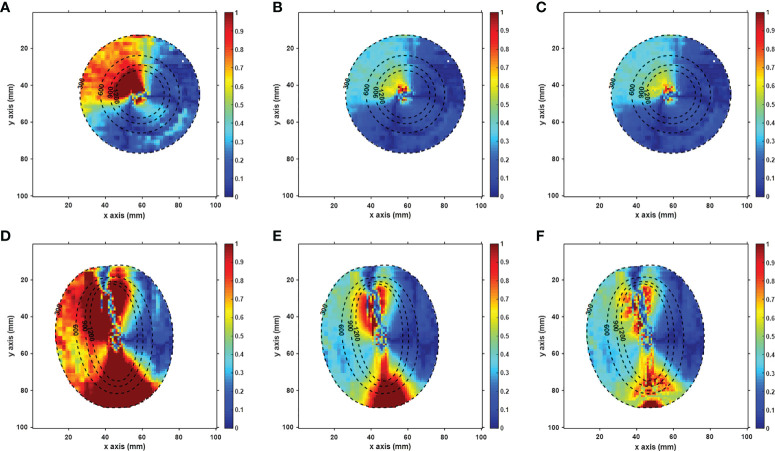
Axial **(A–C)** and sagittal **(D–F)** views of the gamma-index map of the RD_cor. In the gamma calculation, the distance and dose criteria were set to (1%, 1 mm) in a and d figures, (2%, 1 mm ) in b and e figures, (2%,2 mm) in c and f figures, respectively, and the voxels with doses less than 3 Gy were not included. The dotted lines indicate the dose contour (unit: cGy).

**Figure 5** displays the γ-index map under three different criteria, with γ pass rates listed in **Table 3**. Using the criterion (1%, 1 mm), significant dose differences were observed in RD_cor at each dose level. Quite a few high-dose voxels (≥9 Gy) failed the γ-index test, as shown in **Figures 5A, D**. After relaxing the dose criterion to 2%, as observed in **Figure 5B**, most voxels at both ends of the applicator passed the γ-index test (**Figure 4E**). Quantitative analysis revealed that RD_cor achieved a γ pass rate of >97%, indicating significant improvement in the dose calculation accuracy after scatter correction. Furthermore, if the criteria were set to (2%, 2 mm), only scattered high-difference voxels were shown in the γ-index map.

**Table 3:** γ pass rates for RD_cor for the phantom. The second column indicates the distance and dose difference criteria.

Threshold (Gy)		3	6	9	12
Pass rate of RD_cor (%)	1%, 1 mm	87.04	81.63	75.44	71.44
	2%, 1 mm	98.43	98.22	97.85	97.34
	2%, 2 mm	99.63	99.40	99.14	98.84

### 3.2 Patient study

#### 3.2.1 CBCT image quality

**Figure 6:**
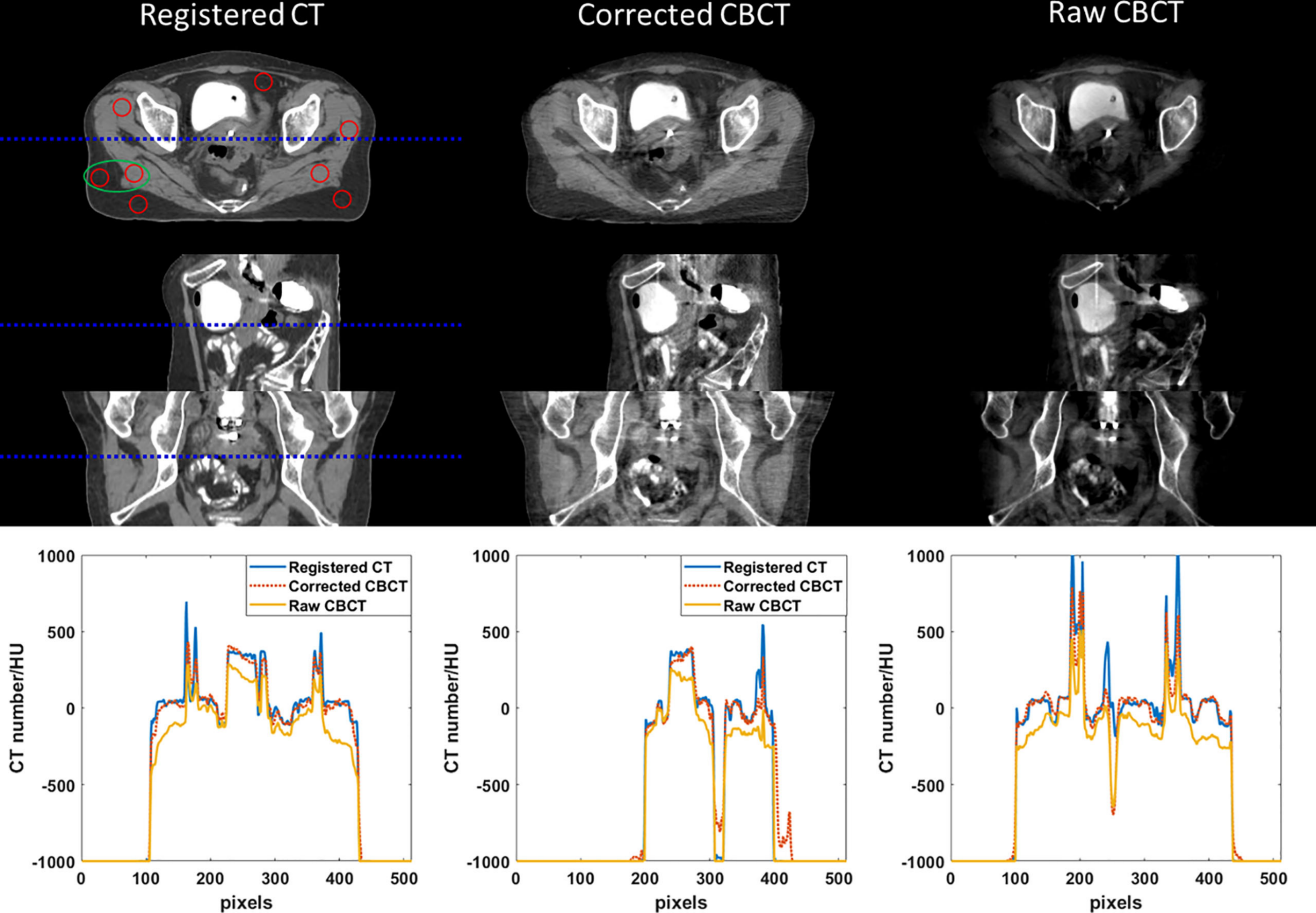
CT/CBCT images of one BT patient. The first three rows display the coronal, sagittal, and axial views from top to bottom; display window: [−200 300] HU. The 1D profiles indicated by the dotted lines are displayed in the last row. The mean CT number and SNU are calculated in the circle areas, and the CNR is calculated in the ellipse area.

**Figure 7:**
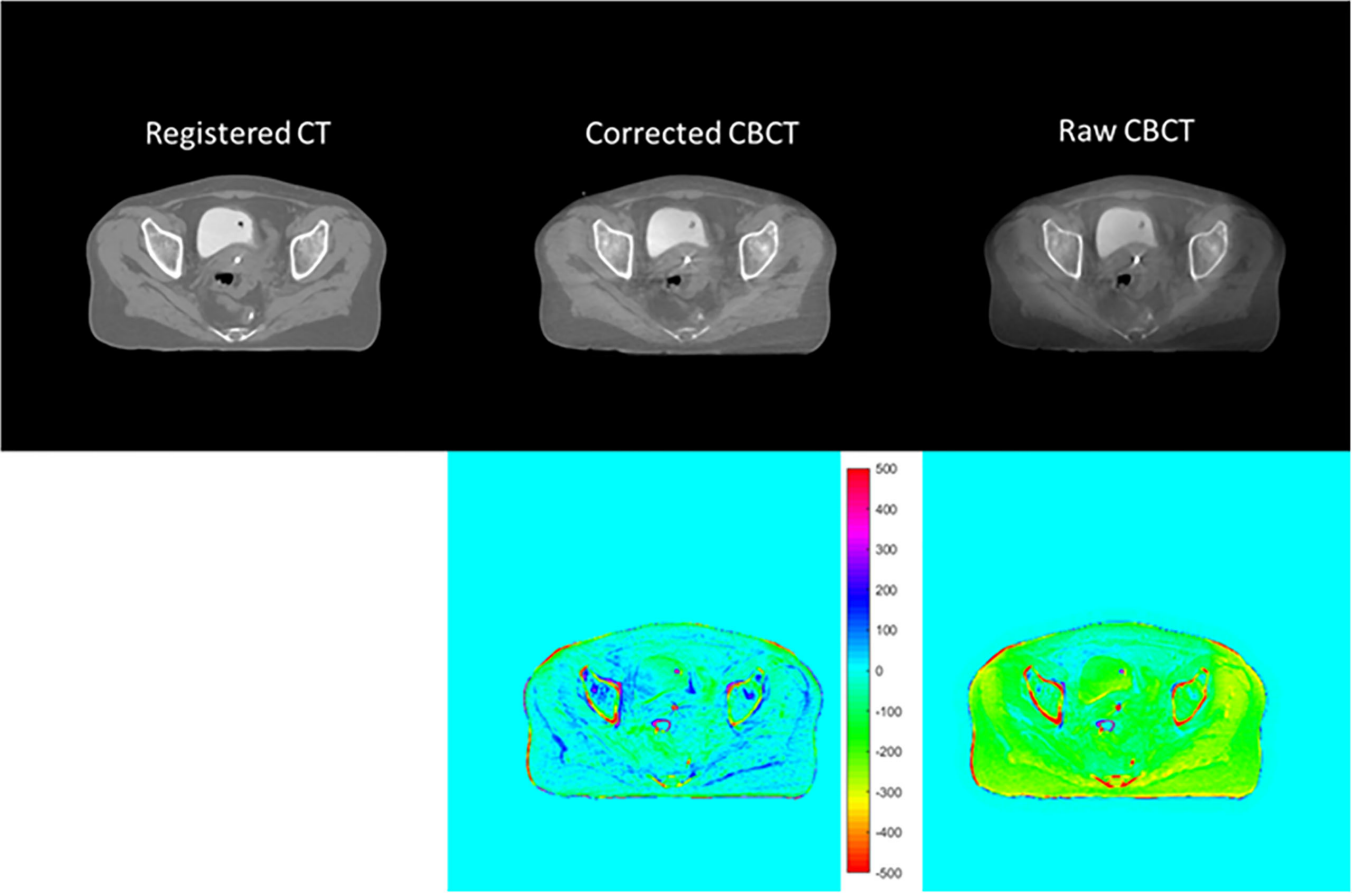
CT/CBCT images of the same patient in **Figure 6** in the axial view and the CT number difference map of CBCT and CT; display window: [−500 500] HU.

**Figures 6** and **7** show the comparison views of a BT patient’s images, with the CT number of this case in **Table 4** and the quantitative analysis for five patients in **Table 5**. As shown in the absolute error images, the raw CBCT suffered a large CT number error around the patient margin, whereas the CT number of muscles around the cervix still had an error of more than 70 HU. **Figures 6** and **7** show that the proposed correction not only compensated for the lower CT number of the marginal tissues but also improved the brightness of the central tissues to a level comparable to rCT.

**Table 4:** The CT number comparison of the eight ROIs in **Figure 6** (unit: HU).

	Registered CT			Corrected CBCT				Raw CBCT			
		CT #	STD	Range	CT #	STD	Range	HU error	CT #	STD	Range	HU error
ROI-1	46	7	(15, 63)	51	14	(10, 82)	5	−102	17	(−149, −71)	−148
ROI-2	44	10	(3, 69)	48	17	(−14, 93)	4	−146	26	(−204, −83)	−190
ROI-3	46	8	(2, 68)	48	17	(−8, 90)	3	−196	9	(−230, −173)	−242
ROI-4	40	10	(−27, 61)	46	15	(0, 97)	6	−213	13	(−239, −182)	−253
ROI-5	−105	9	(−126, −77)	−104	19	(−152, −60)	1	−282	17	(−316, −245)	−178
ROI-6	−104	9	(−128, −79)	−101	13	(−138, −66)	3	−325	11	(−356, −286)	−221
ROI-7	−106	10	(−132, −75)	−99	15	(−138, −55)	7	−319	9	(−344, −291)	−213
ROI-8	−101	12	(−124, −43)	−104	11	(−133, −52)	−3	−138	10	(−164, −98)	−37

**Table 5:** Quantitative analysis of CT number error, CNR, SNU, and RMSE of five patients.

	CT number error/HU		SNU/HU		RMSE/HU	CNR
		Muscle	Adipose	Muscle			Adipose
Registered CT	N/A	N/A	15 ± 5	12 ± 8	N/A	11.32 ± 2.80
Corrected CBCT	4 ± 2	4 ± 3	12 ± 4	12 ± 4	5 ± 1	6.65 ± 1.29
Raw CBCT	200 ± 63	154 ± 66	105 ± 56	154 ± 57	188 ± 25	3.80 ± 1.74

In the patient study, the proposed method achieved similar CT number accuracy as the phantom study, and the error was limited to below 10 HU in each selected ROI. The RMSE of scatter-corrected CBCT was decreased to 5 ± 1 HU from 188 ± 25 HU in the raw CBCT, and SNU on adipose tissue and muscle was both reduced to ≤25 HU from ≥100 HU, indicating significantly improved image uniformity, and the CNR between adipose tissue and muscle was increased by a factor of 1.75.

#### 3.2.2 Dose calculation precision

Using RD_CT as reference dose distributions, average γ pass rates with three different criteria for RD_cor of five patients are listed in **Table 6**. There are lower γ pass rates for high-dose thresholds under the same criterion. Except for the highest dose threshold of 12 Gy, the γ pass rate was >90% for all dose thresholds with the criterion (2%, 1 mm). This means that the further away from the radioactive source, the smaller the difference between RD_CT and RD_cor. Moreover, the RD_cor of patients could realize a γ pass rate of >93% with the criterion (2%, 2 mm) for different dose thresholds.

**Table 6:** Average γ pass rates for RD_cor of five patients. The second column indicates the distance and dose difference criteria $\left(\bar{x}\;\pm \;s\right)$ .

Threshold (Gy)		3	6	9	12
Pass rate of RD_cor (%)	1%, 1 mm	77.32 ± 14.26	74.81 ± 13.91	70.84 ± 12.32	65.92 ± 10.63
	2%, 1 mm	93.60 ± 8.70	93.21 ± 6.54	90.69 ± 5.34	87.19 ± 4.85
	2%, 2 mm	98.19 ± 2.00	96.96 ± 2.23	95.24 ± 2.20	93.07 ± 2.28

**Table 7** shows the dosimetric comparison of HRCTV and OARs based on different images. For both RD_CT and RD_cor, the D_90_ of HRCTV meets the dose requirements, while the D_2cc_ of OARs is below the threshold. Moreover, the average percentage deviation (APD) in these dosimetric parameters between RD_CT and RD_cor is<1%.

**Table 7:** Dosimetric comparison of HRCTV and OARs between RD_CT and RD_cor plans (unit: Gy; $\bar{x}\;\pm \;s$ ).

	HRCTV D_90_	Bladder D_2cc_	Rectum D_2cc_	Intestine D_2cc_	Sigmoid D_2cc_
RD_CT	5.54 ± 0.19	4.58 ± 0.88	3.89 ± 0.32	3.35 ± 0.49	3.19 ± 0.88
RD_cor	5.54 ± 0.16	4.55 ± 0.93	3.88 ± 0.34	3.35 ± 0.49	3.18 ± 0.86
APD (%)	0.01 ± 0.57	−0.83 ± 1.87	−0.37 ± 1.40	−0.01 ± 0.21	−0.26 ± 0.65

APD, average percentage deviation.

### 3.3 Computational efficiency

The CBCT reconstruction with scatter correction was implemented on a personal PC installed with NVIDIA RTX 2080ti GPU and Intel i9 9900k CPU, and the dose calculation was performed on the Intelligent cloud platform (Varian Medical Systems, Palo Alto, CA). The CBCT reconstruction took less than 30 s for 200 slice images using 656 projections with 1,024 * 768 pixels. Using the LBTE solver rather than Monte Carlo simulation, MBDC in the phantom study was completed within 2 min, while in the patient study, the time was increased to 8 min due to the process of volume optimization in inverse planning.

## 4 Discussion

IGBT has proved to be highly effective in cervical cancer treatment. Despite great success, the accuracy of dose delivery still suffers from anatomical variation and applicator displacement due to the transfer of the patient and the long treatment process. To complete image acquisition and treatment in a single room, CBCT guidance was introduced for cervical BT, which has the potential to avoid patient multiple transfers and CT scans, leading to a much simplified treatment process. In this work, we completed image acquisition and treatment in a single room, with the dose calculation finished in 10 min. Additionally, to facilitate accurate organ contouring and MBDC, a hybrid-domain scatter correction using EBRT-CT as a prior was implemented to improve the HU accuracy and soft-tissue contrast.

Prior-information-based methods have advantages in accurate scatter estimation without extra patient dose (29) or hardware modification. These methods usually generate low-frequency scatter signals in the projection domain and require two successive CBCT reconstructions. The proposed scatter correction abandoned this commonly used strategy; instead, the constant scatter-corrected CBCT image was scaled by a low-frequency scatter ratio to remove scatter-induced artifacts in the image domain. Although this simplification inevitably sacrifices the HU accuracy, especially on the high-density bones, the proposed method is still suitable for CBCT-based BT. Due to the rapid dose fall-off, the BT dose was mainly concentrated within 5 cm around the radiation source, an area that is basically soft tissues. On the soft tissues, the proposed methods achieved high HU accuracy and could faithfully reflect the tissue heterogeneity; thus, the corrected CBCT images are accurate enough for MBDC. Moreover, the proposed method could be accelerated using GPU, making the time to acquire BT images much lower than ~31 min (9) currently needed.

The feasibility of performing model-based dose calculations on corrected CBCT was verified in the phantom studies *via* γ analysis. Under each γ-index criterion, the γ pass rates decreased as the threshold dose increased. This phenomenon indicates that voxels with a large percentage dose error are mostly in the high-dose areas, which is consistent with the colored distributions in the γ-index map and may be caused by the slight applicator displacement between rCT and CBCT scans. When using the criterion of (2%, 1 mm) to (2%, 2 mm), the γ pass rates were all >97% in the *CBCT_fc_*-based dose distribution, which revealed that the dose error of most voxels was limited to less than 2%. The patient study illustrated that the γ pass rates were all >93% with the criterion of 2% and 2 mm for both the high and lower dose thresholds. In *CBCT_fc_*-based BT plans, the tumor target received almost the same dose as CT-based BT plans, while there was no additional dose to OARs. These results suggested that in the current clinical situation, the CBCT-guided BT could provide an optional solution for radiation therapists.

The proposed CBCT-guided BT can be further improved in some aspects. First, more patient cases could be studied to fully evaluate the stability of this method. The second is to investigate other scatter correction techniques, such as beam blocker-based (14) and primary modulation-based (22) methods, which may obtain accurate scatter estimation by combining hardware measurement and software processing. In addition, due to the lack of scatter-free and registered images, this study did not investigate the accuracy of dose calculation on the patient CBCT images. Future work will focus on creating scatter-free CBCT images *via* Monte Carlo simulation (20) and evaluating the patient dose accuracy. Currently, the CBCT scan and corresponding reconstruction take less than 90 s, and the dose calculation takes approximately 8 min; however, most of the time before treatment is spent on the manual organ delineation as indicated in Refs (9, 10).. To promote fast and accurate cervical BT, automated organ segmentation (35, 36) dedicated to cervical cancer should be developed and plugged into the workflow of BT.

## 5 Conclusion

In conclusion, scatter correction using planning CT (pCT) prior largely promoted the CBCT image quality, and a dosimetric study demonstrated the feasibility of using corrected CBCT for model-based BT dose calculation. This technique made full use of the pCT scan in EBRT and achieved an error of<15 HU without an extra CT scan or hardware modification. The accuracy of MBDC was also improved after scatter correction as indicated by the increased γ pass rate for prescription dose (>95%, criterion: 2%, 2 mm). Moreover, CBCT-based BT saved the patient transfer and setup to simplify BT treatment, while the real-time imaging avoided applicator displacement and organ deformation to facilitate more accurate dose delivery. Therefore, the proposed CBCT scatter correction and CBCT-based BT have promising prospects in cervical cancer radiotherapy.

## Data availability statement

The original contributions presented in the study are included in the article/supplementary material. Further inquiries can be directed to the corresponding author.

## Ethics statement

This is a retrospective study that analyzed data relating to previously treated patients retrospectively. None of the dose calculations involved in this study were performed on real humans.

## Author contributions

ALW and LZ contributed to the study design and experiments. ALW, HC, and JX completed the data analysis and drafted the manuscript. YB helped with the phantom design and the use of software. ADW and YQL collected the data and evaluated the brachytherapy plans. All authors contributed to the article and approved the submitted version.

## Funding

This research was supported by the National Natural Science Foundation of China (Grant Nos. 11805198 and 81671681), the Ministry of Science and Technology of China Key Research and Development Projects (Grant No. 2016YFC0101400), and the Fundamental Research Funds for the Central Universities (Grant No. WK2030040089).

## Conflict of interest

The authors declare that the research was conducted in the absence of any commercial or financial relationships that could be construed as a potential conflict of interest.

## Publisher’s note

All claims expressed in this article are solely those of the authors and do not necessarily represent those of their affiliated organizations, or those of the publisher, the editors and the reviewers. Any product that may be evaluated in this article, or claim that may be made by its manufacturer, is not guaranteed or endorsed by the publisher.
